# Heritability and Genetic Correlations of Fear-Related Behaviour in Red Junglefowl–Possible Implications for Early Domestication

**DOI:** 10.1371/journal.pone.0035162

**Published:** 2012-04-19

**Authors:** Beatrix Agnvall, Markus Jöngren, Erling Strandberg, Per Jensen

**Affiliations:** 1 IFM Biology, Division of Zoology, Avian Behavioural Genomics and Physiology Group Linköping University, Linköping, Sweden; 2 Department of Animal Breeding and Genetics, Swedish University of Agricultural, Sciences, Uppsala, Sweden; Australian Wildlife Conservancy, Australia

## Abstract

Domesticated species differ from their wild ancestors in a number of traits, generally referred to as the domesticated phenotype. Reduced fear of humans is assumed to have been an early prerequisite for the successful domestication of virtually all species. We hypothesized that fear of humans is linked to other domestication related traits. For three generations, we selected Red Junglefowl (ancestors of domestic chickens) solely on the reaction in a standardized Fear of Human-test. In this, the birds were exposed for a gradually approaching human, and their behaviour was continuously scored. This generated three groups of animals, high (H), low (L) and intermediate (I) fearful birds. The birds in each generation were additionally tested in a battery of behaviour tests, measuring aspects of fearfulness, exploration, and sociality. The results demonstrate that the variation in fear response of Red Junglefowl towards humans has a significant genetic component and is genetically correlated to behavioural responses in other contexts, of which some are associated with fearfulness and others with exploration. Hence, selection of Red Junglefowl on low fear for humans can be expected to lead to a correlated change of other behavioural traits over generations. It is therefore likely that domestication may have caused an initial suite of behavioural modifications, even without selection on anything besides tameness.

## Introduction

The process of domestication, a fast and far-encompassing evolutionary process, started about 15,000 years ago with the dog being developed from the wolf [1], [2]. Since then, many additional species have successfully undergone the same process, including all the common farm animals and the chicken. It is largely unknown how the domestication of animals started, but regardless, a low degree of fear for humans must have been a central trait selected upon already during the earliest periods of the process [3]. In addition, the ancestors of all species that have undergone the domestication process share a number of other traits, such as living in social groups with a hierarchal group structure, promiscuous mating, precocial young, and usually being either herbivorous or omnivorous [4]. During selection, these traits have changed in many ways, causing differences to emerge between the wild ancestors and the present-day domesticated type [5].

Based on experiments on farm foxes, it has been suggested that the reduced fear of humans may drive the emergence of domesticated phenotypes [3], [6], [7]. Belyaev and co-workers selected farmed silver foxes (*Vulpes vulpes*) strictly on low scores in a fear of human test, described in [3]. Many of the morphological and behavioural differences commonly recognized between domesticated animals and their wild ancestors, often referred to as the “domesticated phenotype” [5], [8], [9], spontaneously developed as an apparent side-effect of the selection. This included, for example, an increased frequency of floppy ears, piebald marks, short and curly tails, and changes in reproductive physiology. Hence, it is possible that correlated selection responses may explain the coherent emergence of similarities in different phenotypes of domestic animals, perhaps as a result of the genetic architecture of these traits [10]

In order to study such possible correlated selection responses, we focus in this paper on the chicken. The domestication of chickens started around 8000 years ago [11] in South and Southeast Asia [12]. The species of origin is mainly the Red Junglefowl (*G. gallus*) [11], [13], [14], although the Grey Junglefowl (G. sonneratii) has contributed to some extent as well [15]. Behaviourally, the similarities with the Red Junglefowl are still striking, although the common domestication-related differences are readily identifiable [16]–[20]. In the wild, Red Junglefowl are strikingly wary and fearful [21]. They perch high up in trees as an anti-predator behaviour [21], whereas domesticated chickens perch to a lesser extent [22]. Hence, general fear levels have changed substantially during domestication [23], and this may possibly have affected other aspects of the behaviour as well by means of correlated responses.

In accordance with the assumptions underlying this study, traits such as egg production and growth in modern poultry appear to be linked to fearful behaviours [17] by means of the genetic architecture, so highly productive animals are less fearful. Selection responses in fear has been studied in a number of different species, for example quail [24], rats [25], foxes [3] and mink [26], and in chickens, we have earlier shown that the fear reaction towards humans differs significantly between domestic birds and the ancestors [23], and that variation in fear levels in Red Junglefowl are related to different brain gene expression profiles, i e, groups of genes are up- or down regulated in birds showing higher fear [27].

Thus, studies suggest the hypothesis that early selection of animals with a low fearfulness towards humans may simultaneously affect other behaviours and phenotypes through genetic correlations. In the present study, we studied the heritability and genetic correlations of a number of Red Junglefowl behaviours relevant from a domestication perspective. Starting from an outbred laboratory population of n = 99, birds were grouped and selected based on their level of fearfulness towards humans, as measured in a standardized test. For three generations, birds were additionally phenotyped in a series of behavior tests and their growth and reproduction characteristics were measured. We hypothesized that groups of birds differing in their fearfulness towards humans would also differ in other behaviour phenotypes, and that there would be significant heritabilities associated with the phenotypes, as well as significant genetic correlations between various traits.

## Materials and Methods

### Ethical note

The experiments were conducted under license from the ‘The Linköping regional committee for ethics in animal research’, approval number 122-10.

### Animals, breeding and housing

**Figure 1:**
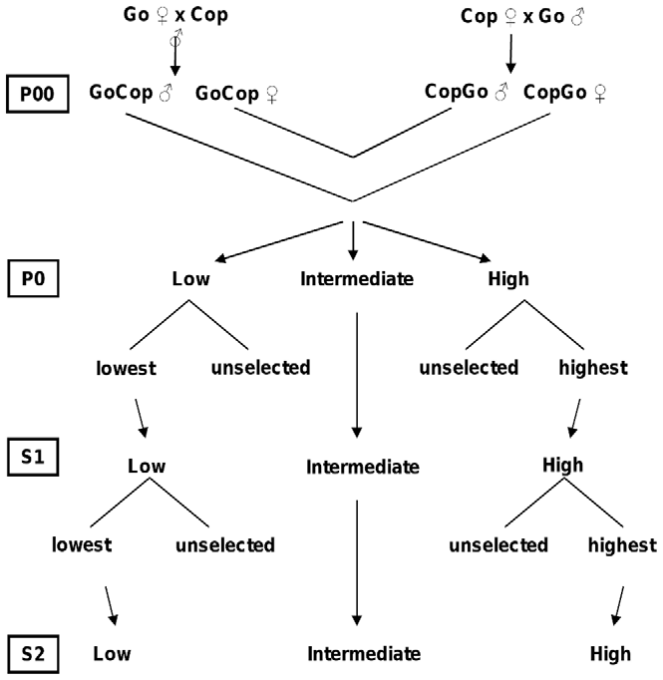
Outline of the selection schedule. From the outbred Copenhagen (Cop) and Götala (Got) population to the second selected generation S2.

The Red Junglefowl used for the experiment originated from two captive populations, which had been maintained in the research facility for more than five generations before the start of the experiment with a population size of about 70–80 individuals per population and generation. The two populations originally came from Copenhagen Zoo (Cop) and Götala research station (Got). They differed significantly in their fear responses, and full information about the background of them can be found in [28]. In order to create the outbred parental (P0) generation, the two populations were first crossbred through two generations, first by mating 13 pairs of Cop females and Got males (Cop×Got) and 15 pairs of Cop males and Got females (Got×Cop). In the next generation, the animals were further outbred by mating 11 pairs of Got×Cop females with Cop×Got males and 8 pairs of Got×Cop males with Cop×Got females. The offspring of this (the third outbred generation) constituted the parental generation of the project (P0). The breeding scheme is outlined in Figure 1, and was designed to obtain as much genetic variation as possible in P0, followed by directional selection for high or low fear in the subsequent generations.

Starting in the P0-generation, all birds were observed in a fear-of-human test at 12 weeks of age (described below). Based on the results, they were divided into a high (H), low (L) or intermediate (I) group. The H and L group each consisted of 27% of the birds with the highest and lowest fear score respectively, and the I group consisted of all the rest.

P0-birds were mated and bred in random pairs within the three groups; 24 low, 24 high and 12 intermediate birds respectively. All their offspring in S1 (first selected generation) were again tested in the same fear-of human test at the corresponding age. Continued breeding of S2 was kept within the three groups, so the 20 most fearful of the S1 H group were bred in random pairs, as well as the 22 least fearful of the L group, whilst keeping strict control of family belonging for each bird. Within the I-group, birds were bred in 11 random pairs (22 individuals). In S2, 14 families of the originally 17 were represented. There were in total 31 H-birds, 60 L-birds and 42 I-birds in S2. Hence, the selection pressures were similar in the two selected groups.

All eggs were incubated in a Marsalles 25 DIGIT incubator, set on 37.5°C, 55% relative humidity, and egg rotation every hour. At day 17, the eggs were placed family-wise in wire-mesh compartments, rotation was turned off and temperature was increased to 37.8°C and humidity to 65%.

After hatching on day 21, all birds were individually wing-tagged, weighed and vaccinated against Marek's disease. They were kept in small pens (0.75×0.75 m) in groups of about 30 birds each with heat lamps and ambient room temperature at about 27°C. The pens were doubled in size at two weeks of age, and at five weeks, all birds were moved to the chicken research facility “Wood-Gush”, situated about 10 km from the university. There, the birds were kept in sex separated groups in pens, measuring 3×3×3 m, containing food and water, nests, perches and wood chips on the floor.

### Behavioural tests and phenotyping

Starting from P0, all birds in each generation were exposed to a number of behavioural tests, described below, and phenotypes relating to growth were collected.

### Fear of humans (FH)

At 12 weeks of age the fear response towards humans was tested. The test was carried out in an arena measuring 100×300×210 cm with solid walls 50 cm up from the floor, and wire mesh above. The floor of the arena was made of concrete and divided into 3 equally sized zones. The bird was placed in darkness on the floor in the middle of the arena and a test person was standing at one short end of the arena. The test started when the light was turned on and the behaviour (according to Table 1) of the chicken was scored with one-zero sampling every 10^th^ second during one minute. Then the test person moved and took position at the second zone in the arena while continuing to score the behaviour of the chicken during the subsequent minute. This was then repeated for the next zone. At the end of the test the person touched the chicken and scored the behaviour according to the same ethogram (Table 1). The fear level of the animal was assessed on a scale from 20–100 at every sampling point (see Table 1), where 20 signified a fearless animal and 100 a highly fearful reaction. After the test, a total fear score was assigned to an individual as the average score of all the sampling points during the test.

**Table 1:** Selection criteria for FH.

Fear level	Behaviour
**20**	Exploring, standing or walking, with short neck.
**40**	Standing or walking with eyes open and neck stretched. Headflicks and vocalizing 1–5/10 sec.
**60**	Standing or walking with eyes open and neck stretched. Headflicks and vocalizing 6–15/10 sec.
**80**	Standing or walking with eyes open and neck stretched. Headflicks and vocalizing >15/10 sec.
**100**	Escape attempts and vocalizing loudly alt. the bird is completely still (freeze behaviour)

The selection criteria for the behaviour in the “Fear of Human”-test, where 20 defines a calm animal and 100 a highly fearful animal.

In order to estimate the consistency in the reactions of the birds, and the inter-observer reliability, ten randomly chosen animals in the S2-generation were tested twice with a five-day interval between by two different observers. The Spearman rank correlations of the ratings of the two observers were calculated, as well as the correlations between the assessments of the birds between the test instances. For the selection only the score from the first performed test were used.

The test generated a fear-score for every individual based on the criteria in Table 1, and, the birds were classified as belonging to one of the three categories described above (H, I, L).

### Social reinstatement (SR)

At three weeks of age, the chicken's sociality was measured in a standardized SR-test. The arena for this behaviour test was a runway measuring 20×120×40 cm. At one short end, two stimulus animals, unfamiliar to the test bird but of same breed and age, were kept in a small compartment (20×40×40 cm) separated from the arena with wire mesh. The stimulus birds were changed after three tests. A social zone (20×40 cm) was defined closest to the social companions.

The test procedure was as follows: the test animal was placed opposite to the social zone in darkness and the test started when the light was turned on. For five minutes the movement of the animal was recorded with a video camera and using the software EthoVision (Noldus, version 3.1). The animals were tested one by one and all the animals were subjected twice to this test, with one day in between. We scored the total time the chicken spent in the social zone (SRSocial) and the total distance it moved during the test (SRDist). The average of the variables from the two test occasions was then used for analysis.

### Open field (4 weeks) (OF4)

In order to assess anxiety and exploration related behaviour [29] the animals were exposed one by one to an open field test at the age of four weeks. While in darkness, the bird was placed in a novel arena measuring 80×120×40 cm, made out of plywood covered with net. The arena was divided into two areas; center (40×80 cm), and periphery. During five minutes, the movements of the chicken were recorded with a video camera, again using the software program EthoVision (Noldus, version 3.1). The test was repeated two times for every individual. Variables recorded were the total distance moved (cm) (OF4Dist) and the proportion of time the chicken spent in the periphery (OF4Periphery). The average of the variables from the two test occasions was used for analysis.

### Foraging and exploration test (FE)

At the age of 13 weeks, the propensity to forage and explore different food sources was tested in an exploration/foraging behaviour test. The arena for the foraging/exploration test consisted of a 0.9 m^2^ square which was separated with wire mesh from an area surrounding the arena (2.7 m^2^), where four companion birds were kept without access to food or water during the tests. Four cardboard boxes (1–4) were taped to the floor, one in each corner of the square arena, and three plastic cups were fixed into in each of them. One cup in each corner contained wood shavings and 20 mealworms hidden therein, one cup contained freely available, familiar chicken food and one cup contained only wood shavings. The relative position of the different cups within each corner-box was changed in a randomized order between each chicken.

The birds had been exposed to the cardboard boxes and the white plastic cups in their home pens during the week prior to the test, and were also familiar to mealworms. One hour before the beginning of the foraging test, a group of five birds was taken from the home pen and placed in a holding pen where they were deprived of food. After the 60 min food deprivation all five birds were placed in the outer area surrounding the test arena for ten minutes, in order to get the bird familiarized with the test situation. After the habituation time, one bird at a time was placed in the inner part of the arena and its feeding behaviour was recorded during five minutes. The number of pecks in the cup containing hidden food was recorded (FEHidden) as well as the number of changes between the corners (FEChanges). Following the five minutes test period, the focal animal was moved to the surrounding area, the inner square was cleaned, and the cups refilled and rotated in all corner boxes before the onset of another trial.

### Aerial predator (AP)

At the age of 15 weeks, the response to a simulated aerial predator attack response was measured. This test assesses the fear reaction towards an aerial predator, and chickens are known to differentiate their defensive behaviour between aerial and ground-based predators [23]. The animals were placed in an arena measuring 50×150×50 cm. The animals were handled in darkness and the test started when the light was turned on. In order to get a baseline of the behaviour, the animals were first observed undisturbed in the arena for five minutes, using direct recording by an observer hidden behind a screen, and one-zero sampling with 10 seconds interval. After five minutes, a hawk silhouette model made out of plywood slid above, lengthwise over the arena, starting 160 cm above the arena and ending 60 cm above it. The hawk silhouette model was hidden behind black curtains both at the start point and at the end point. The behaviour of the animals was then recorded in the same way as before during five minutes. The frequency of exploration (APExplore), stand alert (APStand) and freezing (APFreeze) behaviour (all defined in Table 2), after the chicken had been exposed to the predator were used for analyses.

**Table 2:** Ethogram of the behaviours recorded in the Aeral predator-test.

Behaviour	Description
Explore	Moving, standing or sitting with eyes fully or partially closed and a relaxed body stance.
Stand alert	Immobile in a standing or sitting posture with eyes open and an alert body stance.
Freeze	The bird is completely still.

### Open Field (16 weeks) (OF16)

At 16 weeks of age, birds were again subjected to an open field test, similar to the one at four weeks, with the difference being the observation technique and the dimension of the arena. The arena measured 190×190×100 cm and the center zone measured 100×100×100 cm whilst the rest of the arena was defined as periphery. Using direct observations, the frequency of which the chicken entered the periphery (OF16Periphery) as well as the frequency of crossed zone borders (as a measure of movement activity) (OF16Crossed) was scored.

### Tonic Immobility (TI)

The tonic immobility reaction is a well established test of fear reactions in chickens, assumed to originally have developed as a defence reaction facing a direct predator attack [27]. We performed this test when the birds were 17 weeks old. The animals were taken from their home pen in darkness and then gently put on their back in a wooden cradle, situated in a dimly lit room nearby their home pen, where they were not able to hear or see their pen mates. The person performing the test kept a soft pressure on the chest of the chicken for 10 seconds. If the chicken stayed in the same position for at least the 5 seconds following release, it was considered to be in tonic immobility. Otherwise, the procedure was repeated maximally two more times. The time until rightening (TIRightening) was recorded and if the birds remained in tonic immobility after 600 s they were assigned the max value, and all birds were returned to the home pens immediately after the test.

### Growth and reproduction

All the animals were weighed at hatch. At 33 weeks of age, the birds were mated with birds from the same selection category with respect to family in individual cages (40×114 cm) as described above. The cages contained perches and nest boxes, food and water ad lib, and full visual and auditory contact with neighbor birds. During 14 days, all eggs laid were stored in 15°C for a maximum of 14 days until they were placed in the incubator.

### Statistical analysis of behavioural and phenotypic values

For every variable, we calculated the within generation mean and standard error separately for each selection group. The effects of sex, generation and selection group and the interactions between them were assessed primarily with ANOVA, using General Linear Models. Normal distribution was determined by the Shapiro-Wilk test and if there were significant deviations, the variables were transformed with log_10_-transformation. For the variables APStand and APFreeze, FEHidden and SRSocial, transformation did not make the variables sufficiently normally distributed, so the non-parametric Kruskal-Wallis ANOVA-test was used. The correlation between the two observers and the two different test times in Fear of Human was calculated by the non-parametric Spearman rank correlation analysis. For TI, the truncated variable time to rightening (s), was analysed with Survival analysis. For all the statistical analysis, Statistica version 9 was used.

### Estimation of heritability, genetic correlations and genetic trends

The following mixed linear animal model was used to estimate variance components needed to calculate genetic parameters:
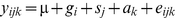
(1)where *y_ijk_* is the observation for the behaviour trait in question (defined under 2.c); *μ* is the overall mean; *g_i_* is the effect of generation (or batch) *i* (*i* = 0, 1, 2); *s_j_* is the effect of sex *j* (*j* = 1,2), *a_k_* is the breeding value of animal *k*, ∼ND(0, **A** 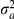), where **A** is the relationship matrix and 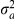 is the additive genetic variance; and *e_ijk_* is the residual ∼ND(0, 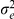), where 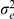 is the residual variance. Heritability was calculated as *h* ^2^ = 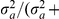 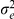). To estimate genetic correlation between traits, a bivariate version of the model above was used with variance components:
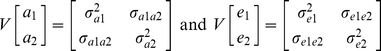
where the subscripts 1 and 2 denote the two traits in question. The genetic correlation was defined as 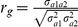


The genetic level for each generation was calculated as the average breeding value of all individuals born in that generation. The average breeding value for generation P00 was by definition set to zero for all traits.

## Results

### Phenotypic effects

The results from the ANOVA-test revealed that generation (P0, S1, and S2) had a significant effect on several of the behavioural variables (Table 3). It should be noted that this generation effect contains not only the genetic change due to selection but also any environmental changes that might have occurred and random noise. For the variables FH, APExplore, TIRightening, FEChanges and the hatch weight, the P0-generation scored higher than the other generations. The middle generation, the S1, diverged from the other generations in APStand, and both variables in OF16. S2 performed more APFreeze behaviour and scored higher in both variables in both SR and OF4.

**Table 3:** P-values for all recorded variables.

	Effects of		
Behaviours	Generation	Sex	Selection
**Fear of human**			
Score (% of max)	<0,001	<0,001	<0,001
**Social Reinstatement**			
Total duration in social zone (s)*	<0,001	0,70	0,15
Distance moved (cm)	0,01	0,88	0,92
**Open Field (4 weeks)**			
Time spent in periphery (s)	<0,001	0,36	0,01
Distance moved (cm)	<0,001	0,19	0,02
**Forage/Exploration**			
Frequency of changes (freq)	<0,001	0,83	0,12
Hidden food (freq)*	0,19	<0,001	0,14
**Aerial Predator**			
Explore (freq)	<0,001	0,61	0,09
Stand alert (freq)*	<0,001	0,37	0,27
Freeze (freq)*	<0,001	0,68	0,41
**Open Field (16 weeks)**			
Frequency of crossed zones (freq)	0,03	0,15	0,60
Frequency in periphery (freq)	<0,001	0,18	0,99
**Tonic immobility**			
Time to rigthening (s)**	<0,001	0,03	0,26
**Weight**			
Hatch(g)	<0,001	0,87	0,02

P-values of the statistical analyses split into generation, sex and selection group. Variables without asterisk were analysed with ANOVA.

* non parametric Kruskal-Wallis ANOVA.

** Survival analysis.

The effect of sex was not as large as the generation effect, but for all three variables that differed significantly between the sexes (FH, TIRightening and FEHidden), females scored higher than males (not shown).

Considering differences between selection lines (H, I, L), there were only significant differences in FH, hatch weight and both the variables in OF4. The H-group scored higher on the FH-test than the other groups whereas the I-group scored highest on the other variables (Table 4).

**Table 4:** Mean (± SE) for all recorded variables.

Behaviours	P0									S1									S2								
	High			Intermediate			Low			High			Intermediate			Low			High			Intermediate			Low		
**Fear of human**																											
Score (% of max)	81,4	±	1,1	66,7	±	0,9	50,4	±	1,3	63,8	±	4,2	53,5	±	6,6	57,5	±	3,6	54,2	±	5,7	51,1	±	3,8	57,7	±	2,5
**Social Reinstatement**																											
Total duration in social zone (s)	40,0	±	4,9	35,5	±	4,1	39,2	±	7,1	52,6	±	8,3	38,5	±	6,9	72,4	±	8,7	211	±	10,2	181	±	8,6	193	±	6,9
Distance moved (cm)	1299	±	86,0	1463	±	107	1496	±	138	1363	±	107	1371	±	167	1269	±	86,2	1733	±	138	1566	±	78,7	1702	±	94,8
**Open Field (4 weeks)**																											
Time spent in periphery (s)	142	±	9,5	157	±	6,5	159	±	11,9	142	±	7,6	182	±	12,7	151	±	7,0	190	±	7,6	199	±	5,6	196	±	5,3
Distance moved (cm)	2220	±	217	3065	±	216	2372	±	305	2357	±	191	3092	±	331	2316	±	142	3177	±	295	3098	±	215	3241	±	136
**Forage/Exploration**																											
Frequency of changes (freq)	5,4	±	1,0	5,0	±	0,8	7,5	±	1,2	3,2	±	0,5	0,8	±	0,3	2,6	±	0,4	3,8	±	0,5	2,7	±	0,4	3,1	±	0,3
Hidden food (freq)	29,0	±	5,5	24,9	±	3,6	27,3	±	4,8	27,6	±	3,5	14,8	±	3,9	18,2	±	2,7	21,0	±	4,2	23,3	±	4,1	20,2	±	2,5
**Aerial Predator**																											
Explore (freq)	8,0	±	1,4	8,7	±	1,1	10,4	±	1,7	3,8	±	0,8	2,5	±	0,6	4,5	±	0,8	4,3	±	1,0	3,7	±	0,6	5,5	±	0,8
Stand alert (freq)	21,7	±	1,7	22,5	±	1,2	15,6	±	1,4	24,6	±	1,2	21,8	±	2,7	23,1	±	1,1	16,3	±	2,0	17,9	±	1,8	18,2	±	1,2
Freeze (freq)	2,1	±	1,3	2,6	±	1,1	6,8	±	2,2	4,0	±	1,2	5,7	±	3,0	4,5	±	1,1	3,8	±	2,1	5,3	±	1,9	1,7	±	0,9
**Open Field (16 weeks)**																											
Frequency of crossed zones (freq)	14,1	±	3,9	16,0	±	3,2	19,3	±	3,5	13,7	±	2,3	10,3	±	5,4	10,5	±	1,4	17,6	±	3,0	20,8	±	3,1	19,1	±	2,6
Frequency in periphery (freq)	5,9	±	2,0	7,3	±	1,6	9,2	±	2,5	5,7	±	1,1	4,2	±	2,3	3,8	±	0,5	7,4	±	1,3	9,4	±	1,3	7,7	±	1,0
**Tonic immobility**																											
Time to rigthening (s)	426	±	40,8	438	±	31,0	330	±	44,9	339	±	39,3	329	±	55,7	324	±	33,8	166	±	38,6	293	±	43,8	294	±	34,6
**Weight**																											
Hatch(g)	27,6	±	0,6	27,3	±	0,4	26,5	±	0,3	24,8	±	0,3	24,9	±	0,5	23,8	±	0,3	21,4	±	0,4	24,1	±	0,5	24,5	±	0,3

Mean (± SE) for all recorded variables within generation for each selection group.

The ratings of fearfulness in the FH test was significantly correlated between the two test instances (rs = 0.89, P<0.05), and between the two observers (rs = 0.83, P<0.05).

### Heritability, genetic correlations and genetic change

The heritability of “Fear of Human”, the variable on which the selection was based, was low (0.17) but significantly different from zero (Table 5). The highest heritability (0.47) was found for hatch weight, but there were also significant heritability estimates for SRDist, as well as for OF4Dist and OF4Periphery. APExplore and FEChanges had heritabilities which were just below significant levels.

**Table 5:** Heritability, genetic correlations and genetic standard deviation.

Behaviours	h^2^			rs			Gen SD
**Fear of human**							
Score (% of max)	0,17	±	0,09*	-		-	5,2
**Social Reinstatement**							
Total duration in social zone (s)	0,06	±	0,06	−0,22	±	0,49	12,7
Distance moved (cm)	0,35	±	0,12*	0,28	±	0,29	371
**Open Field (4 weeks)**							
Time spent in periphery (s)	0,26	±	0,01*	−0,02	±	0,33	23,2
Distance moved (cm)	0,32	±	0,11*	−0,18	±	0,33	694
**Forage/Exploration**							
Frequency of changes (freq)	0,12	±	0,09	0,60	±	0,34*	1,2
Hidden food (freq)	0,03	±	0,06	0,95	±	0,53*	3,3
**Aerial Predator**							
Explore (freq)	0,14	±	0,09	−0,65	±	0,34*	2,2
Stand alert (freq)	0,00	±	0,06	-		-	0,0
Freeze (freq)	0,04	±	0,07	-		-	1,6
**Open Field (16 weeks)**							
Frequency of crossed zones (freq)	0,09	±	0,08	−0,10	±	0,47	4,9
Frequency in periphery (freq)	0,09	±	0,08	0,00	±	0,48	2,2
**Tonic immobility**							
Time to rigthening (s)	0,08	±	0,08	−0,08	±	0,50	65,9
**Weight**							
Hatch(g)	0,47	±	0,08*	−0,09	±	0,25	1,7

Heritability (h^2^) ± SE, genetic standard deviation (Gen SD) of the recorded behaviour variables and the genetic correlation (rs) ± SE between the “Fear of human”-test and the recorded variables.

* signify p<0,05.

**Figure 2:**
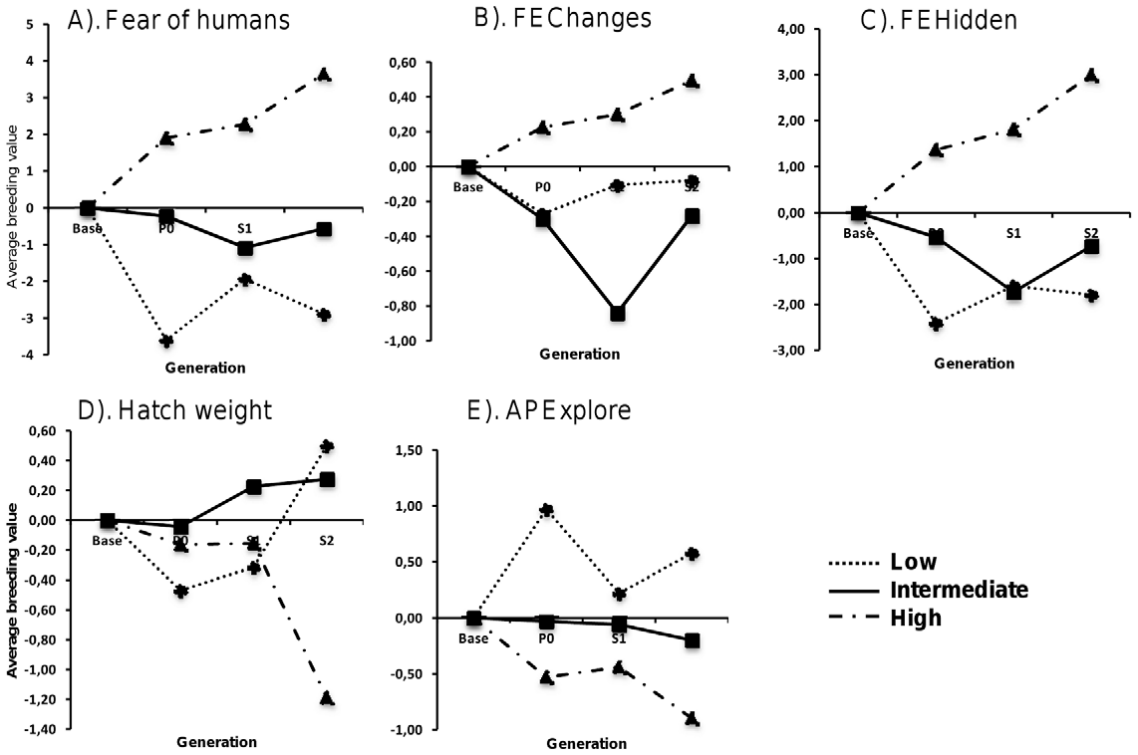
Selection response for the selection lines in each generation. The selection responses measured as average breeding values (BV) for each of the selection lines in each generation for a. Fear of humans, b. FEChanges, c. FEHidden, d. Hatch weight and e. APExplore, BV was by default set to 0 for the parents of the P0-generation.

As shown in Table 5, there was a strong (negative) genetic correlation of FH with APExplore, and moderate correlations with FEChanges and FEHidden. For two variables, it was not possible to obtain a meaningful genetic correlation (AP stand alert and AP freeze), possibly due to the fact that they had very low heritabilities. For all other traits, the genetic correlation estimates were not significantly different from zero. The breeding values for each generation of the variables which had a heritability estimate significantly larger than 0 are shown in Fig. 2. All the variables showed clear trends for a selection response over the selected generations, in particular in the H group. Values for the L group were often overlapping those of the I group.

## Discussion

Our results demonstrate that the variation in fear response of Red Junglefowl towards humans has a significant genetic component. This trait is also genetically correlated to behavioural responses in other contexts, of which some are associated with fearfulness and others with exploration and foraging. Hence, selection of Red Junglefowl on low fear for humans can be expected to lead to a correlated change of other behavioural traits over generations. It is therefore likely that domestication may have caused an initial suite of behavioural modifications, even without selection on anything besides tameness.

It should be noted that the animals in this study originate from populations held in captivity, hence probably already showing a reduced fearfulness in comparison with wild Red Junglefowl. However the project started by two generations of outbreeding in order to maximize the genetic variation of the animals and thereby also the variation in their reaction towards humans. Even though the animals had been held in captivity for several generations, they showed a large variation in tameness. This variation was upheld in the selected generations, in spite of the birds from the three groups being held in the same pens and thereby also experiencing the same day to day experience with humans. So, even if the animals were probably tamer than the pure wild specimens already from the outset of this experiment, the correlations between different traits should not be affected by this.

The three groups of selected animals (H, L and I) differed significantly in traits that are all known to be altered during domestication, i e, hatch weight and anxiety, measured as distance moved and time spent in the periphery of an open arena [30]–[33]. This result already suggests that domestication-related traits may covary with fearfulness against humans. Similar findings have been reported from other species as well. For example, foxes and rats selected on tameness, as well as the domesticated guinea pig compared to their wild ancestors, had lower corticosterone in both serum [30], [34] and feces [25]. This may indicate that the HPA-axis of these animals was less activated and the animals showed a lower overall stress-level. The rats were also less anxious in an open field situation and less fearful overall compared to the wild type [25].

There was a clear selection response for FH, especially for the High line, but also the other lines behaved as expected (Figure 2a). The first generation of selection resulted in a change of 1.9–3.6 units of FH (up or down), which corresponds to 1/3 to 2/3 of a genetic SD. This corresponds well to the expected response from mass selection on a trait with a heritability of 0.17 and a selected proportion of 1/3 (expected response 0.45 genetic SD).

There was also a correlated response to selection in other traits (Figure 2b, c, d), although these were more variable. Nevertheless, there was a clear trend in the expected direction for APExplore, FEChanges and FEHidden.

There was a stronger effect of generation or batch on the phenotype, than there was of selection line. This may be a result of the relatively small population, and could perhaps be attributed to genetic drift between generations. However, it can also be a result of the actual selection regime in the experiment. Traits such as fear and exploration are under control of large complexes of interacting genes [27], [35]–[37], and selecting on one fear trait may in the short term (over few generations) cause large effects on different phenotypes.

Interestingly, the behaviour variables which were most strongly genetically correlated to fear of humans were all associated with fear and exploration. This indicates that these traits will probably be inherited as a suite of behaviours. Selecting on reduced fear of humans will then most likely affect other behaviours related to fearfulness, and to exploration. The correlated behaviours are closely related to aspects of the behavioural complex implicated in the domesticated phenotype. For example, although Red Junglefowl is known to have a higher fearfulness, they are more prone to explore and investigate their environment than domesticated chickens [7], [8], [19], [23]. This is in accordance with the present findings.

Some of the measured behaviours did not show either significant heritabilities, genetic correlations to FH, or phenotypic differences between selection groups. These may represent behaviours which are functionally or genetically unrelated to fearfulness. Perhaps surprisingly, one of the most widely used measures of fear in chickens, the TI response, falls in this category. However, earlier studies have also found that TI may represent a separate and special facet of fearfulness [38], [39] [40].

The mechanisms, which may underlie correlated selection responses like those demonstrated in the present experiment, are as yet unknown. Belyaev [6] suggested that destabilizing selection may explain the complex of domestication phenotypes developed in foxes selected for fear only. According to Belyaev [6] the selection becomes destabilizing when it affects the neuroendocrine control of ontogenesis, for example when animals face new stressful events or environments. The outcome of this would be an alteration in the phenotype of the selected animals, that appears genetically unrelated to the selected character due to a break-up of the previously integrated ontogenetic system [6]. In rats, three possibilities have been suggested to account for the same phenomenon: (1) the traits may be influenced by the genetic variants selected for, i.e. pleiotropy, (2) the traits may correlate because the genes influencing them are situated close to each other, i e, they are linked, (3) the traits may correlate by chance through genetic drift during the selection process.

We suggest that stress related mechanisms may be possible explanations of the correlated effects observed in this study. Stress consists of a physiological response to perceived threatening stimuli, which affects most parts of the metabolism and behaviour of an individual. One may speculate that reduced fear of humans is accompanied by a generally lower stress sensitivity, which is then reflected in many other aspects of the phenotype [6], [41]. For example, the genetic correlation between fear of humans and foraging behaviour found in the present experiment could be a result of this. Red Junglefowl performs more contrafreeloading (explorative feeding) than domesticated chickens [17], [18]. Possibly, this reflects a more energy-consuming strategy of Red Junglefowl, which may be related to an overall higher activity level and higher stress sensitivity. This might be adaptive in the wild, but less so in a captive situation where food and safety is provided by humans.

Although we used a broad variety of fear tests, which would be expected to correlate, they were chosen to reflect different types fear related situations [40] [23] [42]. For example, chickens have different alarm calls depending on whether a predator is encountered from above or on the ground [43] so the aerial predator reaction would not necessarily have to be strongly correlated to the reactions towards humans. Furthermore, the tests also measured other aspects of behaviour, not related to fearfulness, for example foraging and exploration and social reinstatement tendency. The fact that also these aspects were genetically correlated to fear of humans, strongly indicates that the selection we induced affected a broad range of behavioural phenotypes.

In conclusion, we have shown that variation in fear of humans among Red Junglefowl has a significant genetic component, which is responsive to selection. Several behaviours related to fear in other context and to foraging and exploration are genetically correlated to fear of humans and have significant levels of heritability. Hence, it remains a possible scenario, that the necessary increased tameness among the first domesticated chickens was associated with correlated changes in different behaviour systems, even without direct selection on these.
